# Review of Hybrid Fiber Based Composites with Nano Particles—Material Properties and Applications

**DOI:** 10.3390/polym12092088

**Published:** 2020-09-14

**Authors:** Ayyappa Atmakuri, Arvydas Palevicius, Andrius Vilkauskas, Giedrius Janusas

**Affiliations:** Faculty of Mechanical Engineering and Design, Kaunas University of Technology, Studentu 56, 51424 Kaunas, Lithuania; arvydas.palevicius@ktu.lt (A.P.); andrius.vilkauskas@ktu.lt (A.V.); giedrius.janusas@ktu.lt (G.J.)

**Keywords:** natural and synthetic fibers, hybrid composites, thermosets and thermoplastic resins, fabrication techniques, chemical treatment, nanoparticles, applications

## Abstract

The present review article provides an overview of the properties of various natural and synthetic fibers for the fabrication of pure natural composites and the combination of both natural/synthetic fibers-based hybrid composites, bio-based resins, various fabrication techniques, chemical and mechanical properties of fibers, the effect of chemical treatment and the influence of nanoparticles on the composite materials. Natural fibers are becoming more popular and attractive to researchers, with satisfactory results, due to their availability, ease of fabrication, cost-effectiveness, biodegradable nature and being environmentally friendly. Hybrid composites made up of two different natural fibers under the same matrix material are more popular than a combination of natural and synthetic fibers. Recent studies relevant to natural fiber hybrid composites have stated that, due to their biodegradability and the strength of individual fibers causing an impact on mechanical properties, flame retardancy and moisture absorption, natural fibers need an additional treatment like chemical treatment for the fibers to overcome those drawbacks and to enhance their better properties. The result of chemical treatment on composite material properties such as thermal, mechanical and moisture properties was studied. Researchers found that the positive influence on overall strength by placing the filler materials (nanoparticles) in the composite materials. Hybrid composites are one of the fields in polymer science that are attracting consideration for various lightweight applications in a wide range of industries such as automobile, construction, shipping, aviation, sports equipment, electronics, hardware and biomedical sectors.

## 1. Introduction

In recent years, many researchers have been showing an interest in the utilization of natural fibers for the preparation of hybrid composites because of its abundance, low density, recyclability and great strength [1,2], Natural fibers received a great deal of consideration as a potential elective alternative for synthetic fibers [3] as a support of different reinforcement for innovative applications due to their properties, for example, their availability in nature, low cost and they are environmentally friendly. Their resources do not emit any harm full gasses and absorb carbon dioxide, which reduces environmental pollution [4,5,6].

One of the primary drawbacks of using natural fibers as reinforcement in composites is the hydrophilic in nature, which makes them inconsistent with hydrophobic matrices. For refining the inconsistency, the surface treatment of natural fiber is required to advance the global properties of the composite [7,8]. Apart from this, natural fibers are readily accessible to the flame, to overcome this drawback, various fire retardants have been added to them to advance its fire resistance. Surface modifications by using various chemical compounds tend to increase their mechanical and physical properties. And also, structure modification of the composite materials leads to extra stability [9,10].

Over the previous years, there has been an ever-expanding enthusiasm to fuse at least two fillers into a typical matrix. There has been a consistent increment in the quantity of distributed works that are identified with hybrid composites from the past ten years. in view of hybrid composites (between 2010 to 2020) found in the Web of Science and Scopus via looking for the words ‘hybrid composites’ with 26,450 research yields. The development came about because of one goal, to progress the properties of the subsequent composite material to accomplish the wanted properties and improved execution of such composites.

**Figure 1:**
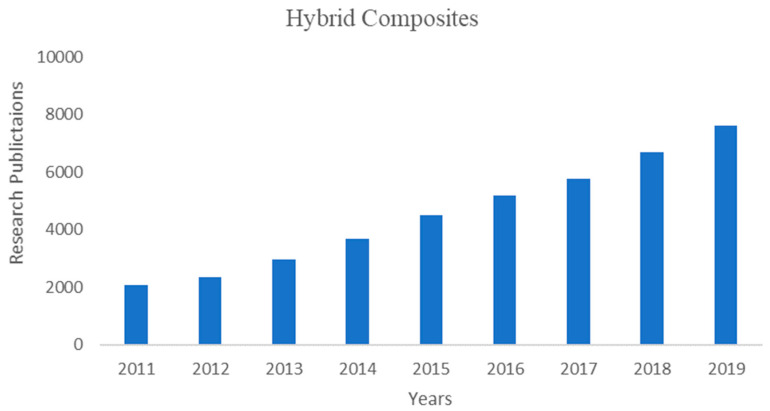
Recent publications on hybrid composites.

Figure 1 shows the total research outcomes on hybrid composites in recent years and the input was from the Web of Science. Most of the research articles are from China, followed by India. The hybridization process intersects the most research articles, for example; material multi-disciplinary, chemical industry, polymeric science, nanoscience, nanotechnology, material science engineering, electrochemistry, mechanics and mechanical engineering so on.

**Figure 2:**
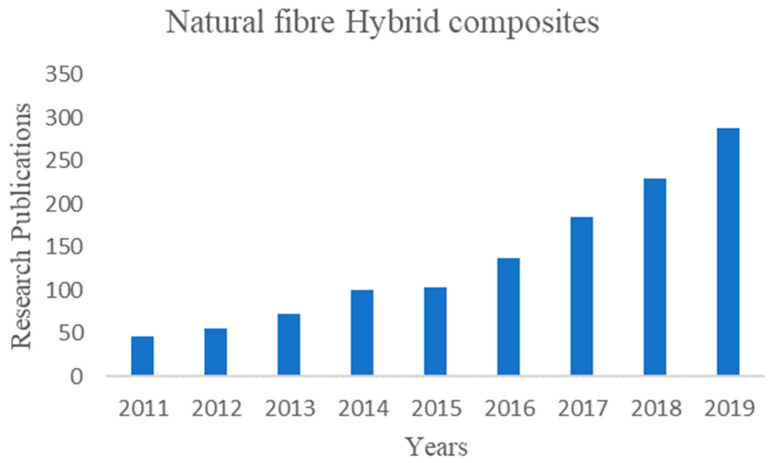
Recent publications on natural fiber hybrid composites.

Figure 2 shows the total research publications on natural fiber hybrid composites. The input was from the Web of Science by searching for “natural fibers and hybrid composites.” There is a total of 1460 research yields, with most of the researchers from material science and polymeric science engineering. The process of the hybridization of natural fiber composites started in early 2008 and then the interest slightly increased. The main aim is to improve the composite properties by adding two or three different filler materials under the same matrix material. Many researchers proved that the orientation of filler material also plays a key role in the composite [11,12,13].

Hybrid Composites are the materials that are made up of twofold or more constituent materials, namely matrix and reinforcement [14,15]. A matrix is a coupling material which binds the reinforcement. Reinforcements are the materials which are embedded in the matrix. These give the additional strength to the composite. The significance of the composites is to provide the desired physical, chemical and mechanical properties in an improved manner, which differs from the individual constituents [16]. Based on the matrix, composites are categorized as polymer, ceramic and polymer. Depending upon the reinforcement these are varied as laminate, fibrous and particulate composites. Depending upon the type of availability, reinforcements are classified as either natural or synthetic [15,17].

If the compound material is metal fiber then it is called a metal matrix composite, if it is a polymer matrix then it is called a polymer matrix material [17,18]. The fiber reinforced polymers (FRP) consist of a polymer matrix and high-quality fibers joined with an unmistakable crossing point among them. In this shape, the two matrices and fibers hold their chemical and physical properties.

## 2. Natural Hybrid Fiber Composites

Natural hybrid composite materials contained fibers of great quality and a modulus introduced in a matrix material. Along these lines, every one of the individual materials (fiber and matrix) holds its chemical and physical properties and they give a combination of properties which would not be conceivable if they were utilized alone. Here, the fibers act as the central load-carrying agents and the matrix material encompasses them in position alongside, acting as a load carrying part between them, protecting the fibers from the natural harms of raised temperature and humidity. Accordingly, it could be properly said that the fiber gives reinforcements to the matrix; thus, the name Fiber Reinforced Composite Materials (FRCM).

Reinforced hybrid materials are made by joining at least two distinct varieties of fibers in a distinctive matrix material. Hybridization of two sorts of filaments with particular lengths and widths offers a couple of central focuses over the use of both of the strands alone in a lone polymer lattice. Most studies are on the hybridization of regular filaments with glass strands to upgrade the properties. They have average calorific regard and cause little stress, similar to prosperity, wellbeing and protecting Also, they show amazing mechanical properties, have a low thickness and are modest [7,14,19,20,21]. The benefits of composite materials by confessional materials, such as greater rigidity and durability, make them more flexible to use. The availability and cost of these natural fibers attract researchers and also have promising results of composite fabrication for real-life applications. Composite materials are comprised of at least two materials with physically detachable phases.

### 2.1. Characterization of Composites

**Figure 3:**
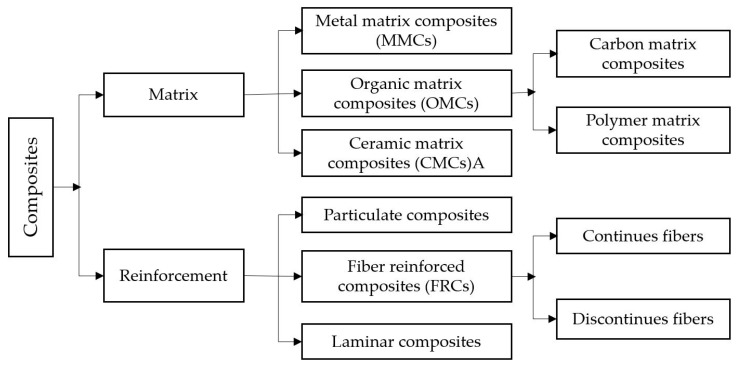
Classification of composites [22].

Composite materials classified on the basis of a matrix serve as a binder and a reinforcement, which acts as a filler material. Figure 3 shows the classification of composites.

### 2.2. Metal Matrix Composites (MMCs)

Metal matrix composites (MMCs) have numerous advantages over solid metals such as great strength, modulus and better-quality properties at raised temperatures. As a result of these qualities, metal matrix composites are getting looked at for a wide scope of applications. On account of their high thickness, they are not greatly requested. The most commonly used metals in the MMCs are steel, tungsten, boric and, molybdenum [22,23].

### 2.3. Ceramic Matrix Composites (CMCs)

Ceramic matrix composites (CMCs) and carbon-based composites are examples of inorganic (non-metallic) composite materials. The main aim of CMCs is to improve the toughness of the composite materials [24] and they are intended to overcome the problems of monolithic ceramics. The problem caused by the matrix materials is less than that of the failure of fiber materials, so it has a great impact on composite fabrication. To avoid the general drawbacks during the processing (early failure), there are several techniques implemented such as gas-phase and liquid-phase fabrication of CMCs.

### 2.4. Organic Matrix Composites (OMCs)

The organic matrix composites (OMCs) are broadly classified into polymer matrix composites and carbon-carbon matrix composites. Polymer composites are the most popular and commonly used matrix materials. The mechanical properties of polymeric composites are easily applicable to a wide range of structures because of their desirable mechanical properties, such as low mass, high stiffness and high-temperature resistance. When compared to the metal and ceramic composites, polymer composites have less strength and stiffness; to overcome this problem, composites are reinforced with other materials. The handling of polymer composites need not include high temperature and pressure. Likewise, the equipment required for the fabrication of composites is more straightforward. Therefore, polymer matrix composites quickly became famous and of interest for many researchers in terms of basic applications. Composites are utilized in light of the fact that the general properties of the composites are better than those of the specific segments.

Polymer composites are further classified as thermosets and thermoplastic composites. Thermoplastic materials are solids at room temperature; heating is required to soften or melt the material to place it into the mold and then it is cured to get the desired shape. These are recyclable. Thermosetting polymers are in both liquid and solid form and these are not recyclable. In thermoplastics, the atoms are bonded by covalent bonds whereas thermosetting polymers have cross-linked bonds, because these thermosetting materials are stronger and stiffer than thermoplastics. Thermoplastic materials include polyamides, polyoxymethylene (POM), polycarbonates (PC) and acrylics. Thermo setting materials include epoxies (EP), thermoset polyamides, amino plastics and polyester thermosets (TS) [24].

### 2.5. Fiber Reinforced Polymer Composites (FRCs)

Fiber Reinforced Polymer Composites (FRCs) are the most widely used composites and are composed of fiber and matrix. Fiber serves as a reinforcement and acts as a stiffness agent. The matrix material glues all fibers together and acts as a stress distributor among the fibers. Sometimes, the filler material is added to smooth the fabrication process. Depending upon the availability, reinforcements are classified into natural and synthetic fibers. Though there are many advantages to synthetic composites, there are some disadvantages. Synthetic fibers burn more quickly than natural fibers. Extra electrostatic charge is produced by rubbing them with natural fibers [23,25].

## 3. Classification of Fibers

### 3.1. Natural Fibers

**Figure 4:**
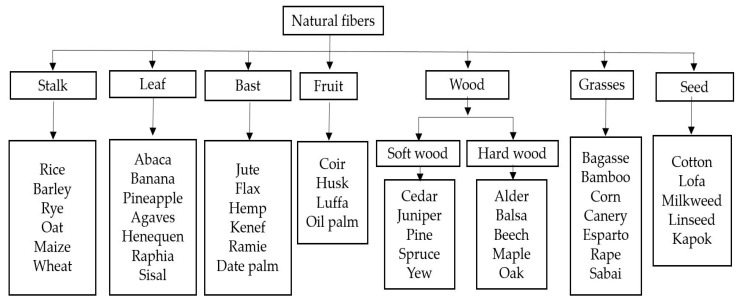
Classification of natural fibers [22,23,24].

Natural fibers are produced from animal (protein is the main component), plant (cellulose is the main component) and mineral based resources. Natural fibers have advanced physical and mechanical characteristics when compared to the other fibers. They are readily available in nature and also biodegradable [26,27]. Because of its lightweight, high strength, low cost and corrosion resistance properties it is drawing attention for use in industrial applications [28]. On the other side, natural fibers have few disadvantages, like high water absorption, high anisotropy, less homogeneity when compared to the synthetic fibers, development of stresses among the fibers in a composite [28]. The following Figure 4 shows the classification of natural fibers. 

The chemical composition plays a significant role in fiber materials and it depends on the fiber source. It varies from fiber to fiber and also within the family as shown in Table 1. Their performance is mainly dependent on the aspect/ratio and cellulose crystallinity of the fibers. Previous studies stated that these fibers are biodegradable because of their chemical constitutes. For example, UV degradation because of lignin and hemicellulose is accountable for thermal and biodegradation and also water absorption. The physical and mechanical properties of the natural fibers are shown in Table 2. Cellulose, hemicellulose and lignin are the main components in the natural fibers. Due to the nature of development conditions, the percentage of these components varies accordingly. To consider the behavior of these fibers, it is important to consider the structure of the components. Cellulose is one of the major building blocks of the natural fibers, it makes up around 50% of plants. The presence of cellulose in the plant fibers has an impact on moisture absorption. Hemicellulose is the second major component followed by cellulose and it is less remarkable as a filler material. Phenylpropane derivatives is comprised of lignin and it is a formless normal polymeric material that controls the transaction of fluid and also combines the hard cells in the plant. Lignin acts as a cementing material and it influences the structure, properties and morphology [29]. Some of the advantages and disadvantages of natural fibers are explained in Table 3.

### 3.2. Synthetic Fibers

**Figure 5:**
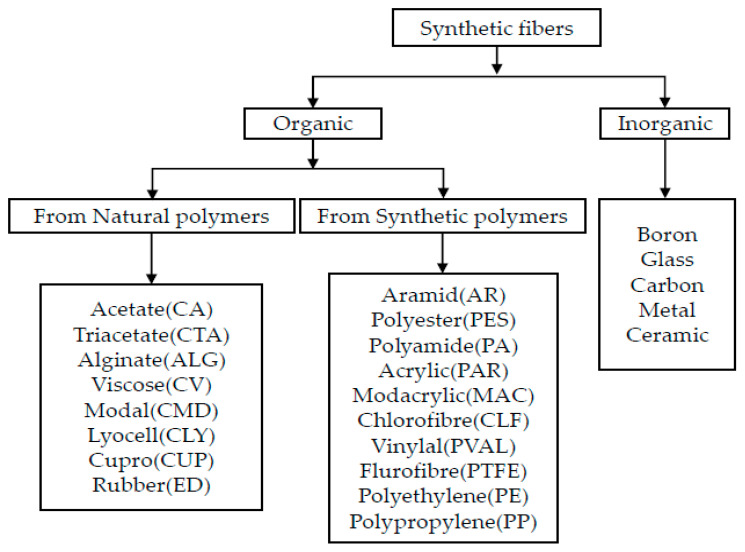
Classification of synthetic fibers [15].

There are various man-made or synthetic fibers that have been brought into recent research networks with respect to their remarkable properties. The mechanical properties of synthetic fibers are shown in Table 4. Among all synthetic fibers, glass and carbon fibers are the most used filler materials in the hybrid composites. These fibers are manufactured by using the polymerization concepts, which includes joining monomers to form a great chain. The merits and demerits of synthetic fibers explained in Table 5. Below Figure 5 shows the classification of synthetic fibers.

## 4. Chemical Treatment of Natural Fibers

Natural fibers are not defect-free materials even though they have great qualities when compared to the other advanced fibers. They have strong polar bonds, which may affect the bonding capability in the polymer matrix [30]. To avoid this, some sort of chemical treatment is required to improve it. Chemical treatment is one that gives additional strength to the composite material to develop a relationship between fiber and matrix material during the curing process. It also improves the surface quality and decreases the amount of water absorbed (Moisture content) by the composite material [31]. Some of these treatment techniques are listed down in Table 6.

## 5. Selection of Matrix Materials for Fabrication

A matrix material is one which plays an adverse role in the composite material. The shape, durability and surface properties depend on matrix (resin) material. Based on processing techniques, resin materials are classified into two types, such as thermosets and thermoplastics [48]. A matrix is used as a load distributer among the reinforcement material when external pressure is applied. Thermoplastics are widely used resin material over thermosets because of its ready pick-up shape to the required mold (moldability). Thermosets are not recyclable because it does not come back to its original state when the resin is converted from liquid state to solid state after the curing process. Only a heating process is required for the thermoplastics to form a new shape.

### 5.1. Thermoplastics

Researchers are showing interest in using thermoplastics as matrix materials because of their integral properties, such as low cost, moldability, less weight and recyclability. The main advantages and disadvantages of these thermoplastic polymer materials are discussed below in Table 7. The viscosity range is more than 500 times that of uncured thermosets during the melting process. Recent results proved that the addition of chemical treatment to the natural fibers may lead to improving the bonding properties between matrix and reinforcement [49], chemical treatments such as; alkaline, silane, acetylation, benzoylation treatments and many others. Due to the addition of chemical and biological treatments, the overall properties of composites are improved [50]. Among all synthetic polymers, thermoplastics are the most commonly used commercial purpose materials. The most widely used thermoplastics are polyvinyl chloride (PVC), polystyrene (PS), polypropylene (PP), polyethylene and natural rubber. The mechanical properties of thermoplastic resins are explained in Table 8.

Kasama et al. investigated the mechanical and thermal properties of sisal and glass fibers as reinforcement and polypropylene as a matrix material [51], the authors reported that the addition of polypropylene to the hybrid composites causes improvement in thermal stability, moisture resistance and also interfacial adhesion between reinforcement and matrix improved. Schmidt et al. reported the mechanical properties of the hybrid composites by using the RTM (resin transfer molding) technique [52]. They analyzed the woven and non-woven composites by in-plane permeability of the hybrid composites and draw a contrast between both of them for comparison purposes and reported that improved mechanical properties and flow media of sisal fibers and glass mats by adding pp to the hybrid composite. Nayak et al. summarized the dynamic mechanical and rheological properties of bamboo glass fiber hybrid composite PP as a resin material by using an injection molding technique [53]. Interestingly, the researcher found that the addition of Maleic anhydride grafted polypropylene (MAPP) in place of normal PP has given advanced results. Vega-Hernandez et al. developed the composites grounded on blue agave bagasse by using polystyrene as a resin matrix [54]. They have invested in the mechanical performance of the hybrid composites by in situ reversible addition-fragmentation chain transfer (RAFT).

Haneefa et al. worked on the tensile and flexural properties of banana and glass fiber reinforced PS composites [55]. It was discovered that the rigidity and modulus of the composite increases by increments in the volume part of the glass fiber. This is expected due to the more noteworthy similarity of glass fiber than banana fiber to polystyrene. In any case, the stretching at break diminishes in increments in the volume portion of glass filaments—this is because of the lower prolongation at the break estimation of glass fiber. Experiments proved that chemical modifications of composites exhibit 30% greater mechanical properties than normal composites. Hao X et al. examined the effects of fiber geometry and alignment on the anisotropy of the flexural and thermal expansion of natural fiber polyethylene hybrid composites [56]. Samples were fabricated by using the extrusion process and, to find out the fiber orientation effectiveness, composites are cut by various off-set angles for comparison purposes and the results showed that at zero angle, composites showed the best mechanical properties whereas at 90 degrees some deviation was observed. Robledo-Ortiz et al. developed green polyethylene natural fiber bio composites by using a rotational molding technique [57]. In this work, composites were prepared by using the rotomolded (rotational molding) technique to develop the bio composites. Natural fibers were combined in a low-density green polyethylene medium and after fabrication, samples were taken for testing mechanical properties. Maleate polyethylene (MAPE) was utilized for surface treatment with the purpose of increasing its adhesion properties. Results indicated that its less elastic nature was because of its poor bonding capacity between reinforcement and matrix. Paran et al. examine the dynamic properties of the green thermoplastic elastomer vulcanizate (GTEV) nanocomposites based on poly vinyl chloride (PVC) and rice straw as reinforcements [58]. The surface morphological studies exposed that the GTEV nanocomposite showed a great surface roughness fracture and specified the presence of some relations between the matrix material and natural fibers [59]. The effect of loading on fibers was foreseen through using the parallel and serious equations [60]. Boussehel et al. worked on the impact of chemical treatment on polyvinyl chloride natural fiber hybrid composites [61]. In this research, the authors drew a contrast between the PVC with and without the chemical treatment of natural hybrid composites and the results showed a slight variation in the mechanical properties [62]. But there is a change of structure for natural fibers after chemical treatment due to the formation of hydroxyl bonds [63]. Haseena et al. carried out research on the interfacial adhesion of natural hybrid fiber natural rubber composites by using the equilibrium technique [64]. The swelling coefficient was found to diminish with an expansion in fiber stacking and penetrant size. The holding operator added blends demonstrated more noteworthy limitation to growth because of the more grounded interfacial grip and the resultant higher cross-linked density [65]. The recent research on various fabrication techniques of thermoplastic resins is discussed in Table 9.

### 5.2. Thermosets

Thermosets are well known for their exclusive uniqueness of making 3-dimensional bonds after curing. Thermoset resin far differs from thermoplastics and these have improved mechanical properties, thermal stability, resistance to the creep and biodegradability [83]. The merits and demerits of some of the thermoset resins are explained in Table 10. Unlike thermoplastics, thermosets are not reusable materials [84]. Epoxy resin, vinyl ester, polyester, phenolic, urea-formaldehyde and many others are some of the examples for thermoset polymers. Among all the resins, epoxy and vinyl ester are widely used resins because of their availability, flexibility and low cost [85]. The primary worry for thermosets is the long length related to handling. Moreover, with the low recyclability of these composites, they require new procedures of structuring maintainable reusing components. For instance, they can be crushed and utilized again as filler materials. The primary target has been the adjustment of the durability of the thermosets and augmenting the handling temperatures. Because of the enhancing mechanical properties as shown in Table 11, thermoset based composites have a huge market for commercial purposes such as the aerospace industry, sports equipment, lightweight objectives, construction field, automobile, biomedical and many other real-life applications. Amongst all these, epoxy resin along with hardener is mostly used in hybrid composite fabrication. There are several processing techniques (Table 12) available in the present time, including the hand lay-up technique, the combination of hand lay-up with compression molding, pultruded, hot press molding, twin-screw extruder and the vacuum assisted resin transfer molding technique (VARTM). The hand lay-up process is mostly used because of cost-effectiveness and easy procedure with improved results. Although there are some drawbacks like curing process, nonuniformity of resin material, vacuum voids in the composite and these are considerably lower compared to the other techniques.

Yorseng et al. developed the bio-based natural fibers hybrid bio epoxy composites for the application of semi-structured components and their mechanical behavior under various circumstances was observed [86]. Surface morphology was conducted by using contact angle measurement properties to find the wettability of the composites. The results showed that a decrease in mechanical properties because of the bio epoxy degradation by accelerated weathering conditions and the ductile nature of the composite are increased and it causes breakdown [87,88,89]. Thermal studies stated that bio-epoxy based natural fiber composites withstand up to 295 °C temperature, which means these composites are valid for high-temperature applications. Alves et al. examined the dynamic mechanical properties of hybridized jute and biaxial glass fiber reinforcement and epoxy as matrix material composites [90]. The vacuum infusion technique was used for the best impregnation level for both fibers during the fabrication process. This paper offered the woven and non-woven fabrics reinforced epoxy resin composites and the effect of fiber arrangement on mechanical properties. Asim et al. investigated the dynamic and mechanical characteristics of Kenaf and pineapple fibers reinforced phenolic hybrid composites [91]. Test samples were prepared by using the hydraulic hot press and fibers were ground up to 0.1 to 0.8 mm and compressed for 8 min to get the desired shape and then used for testing purposes. From the outcomes, it is seen that kenaf composites showed advanced mechanical properties whereas pineapple showed the least stability under phenolic resin but the difference is not very comparative [92]. The wear resistance of natural corn stalk fiber reinforced phenolic resin composites were prepared by using the wet granulation technique [93]. The effect of phenolic resin on the brake friction materials was observed by varying the percentage of resin matrix in the composite weight. Results showed that the wet granulation method improves the surface morphology of the friction materials and also stated that tribological behavior also increased.

Singha et al. worked on the synthesis and characterization of pine needles reinforced resorcinol-formaldehyde (RF) biocomposites [94]. Composite sheets by varying the fiber loadings were fabricated by using the compression molding technique and allowed for mechanical properties testing. Results showed that pine needle composites showed more improved mechanical properties than neat RF resin composites. Composite strength increased along with fiber loadings so it has the potential as a replacement for synthetic fibers. Singha et al. investigated the mechanical, thermal and morphological properties of grewia optiva fiber reinforced resorcinol-formaldehyde polymer composites [95]. Composites were fabricated by using the compression molding technique. Fibers were taken in the form of particles for the fabrication and test specimens allowed for material properties testing and composites were prepared by varying the fiber loadings (by weight). Results showed that an increase in mechanical properties up to 30% fiber loadings and then a slight decrease in it due to weak bonding between matrix and reinforcement material in the composite. Thakur et al. studied the mechanical properties of hibiscus sabdariffa fiber reinforced urea-formaldehyde (UF) green composites [96]. Results suggested an improvement in mechanical properties and there is a huge difference between the fiber composites and neat UF resin composites. Fiber composites are much stronger than pure resin composites. Singha et al. worked on the fabrication and characterization of saccharum cilliare fiber-reinforced resorcinol formaldehyde green composites [97]. Samples were fabricated on a compression molding machine. These test specimens were allowed for mechanical properties testing. Results suggested that saccharum cilliare fibers composite were higher than the matrix composites and these fibers have the potential to be used in composite fabrication.

### 5.3. Bio-Based Resins

Bio-based polymeric resins are produced from renewable sources. These are classified into three types based on their physical properties. The first one is fully bio-based and biodegradable resins those are polyhydroxyalkanoates (PHA) and starch, the second one is partial bio-based resins those are polylactic acid (PLA) and cellulose and the last one is partial non-biodegradable resins those are polyethylene terephthalate (PET) and polyethylene. Most of these bio-based resins are originated from plants and plants-based sugars. The merits and demerits of bio-based resins are discussed in Table 13.

## 6. Integration of Nanoparticles in Natural Fiber Hybrid Composites

Hybrid composites are not defected free materials because of the fabrication errors, voids in the composite, weak bonding between matrix and reinforcement, environmental conditions, errors during the development and curing period, scratches on the fiber materials, impurities, unbalanced proportion of matrix and reinforcement affect the total performance of the composite materials [120]. Limiting the reasons for corrupting components can assist with keeping the presentation of composite materials. The addition of new constituents and material advancement quality control techniques is necessary to progress the performance of the hybrid composites. One of the best ways to overcome these problems is the integration of nanoparticles or nano constituents. Recent reports state that the addition of nanofillers in the composite materials showed greater improvement in mechanical properties such as tensile strength, flexural strength, creep strength, tensile strain, shear modulus, impact strength and also thermal properties than individual polymer-based hybrid composites [121].

Nowadays researchers are using three types of nanofillers to improve the performance of the composite material. They are classified into three main groups such as nanotubes, nanoparticles and nanolayers. Nanofillers are used based on the particle sizes—one dimensional, two dimensional and three dimensional. Nanomaterials are larger than macro-level particles, individual atoms and also have a huge surface area per unit volume. Most of them are produced from metals, carbon, polymers and metal oxides. Nano clay, carbon nanotubes, carbon black and graphene are the best example of metal-based nanofillers and also widely used nanomaterials.

Pappu et al. worked on unused materials, like fly ash and sisal fibers, to prepare the micro and nanocomposites for multifunctional applications [122]. The hand layup technique along with compression molding used for the preparation of composites, fly ash and sisal fibers were used as reinforcement and epoxy used as a matrix material. They prepared a total of two composites fabricated for comparison purposes. Results indicated that the presence of natural fiber and nanofiller boost the performance of the composite, particularly the cellulose content in the sisal fiber and silica along with alumina in fly ash leads to the potential growth of mechanical properties. Majeed et al. reported on food packaging materials from natural fiber hybrid composites, which have nanofiller as nano clay [72] and developed a new method to enhance the bonding between matrix and reinforcement. The addition of nano clay to the hybrid, composite showed good progress in mechanical and thermal properties. But these are not suitable for all types of packaging applications because of higher biodegradability and moisture absorption properties than that of synthetic fibers. Essabir et al. investigated the mechanical and thermal properties of clay and palm fiber hybrid composites [123]. They reported that the hybridization of oil-palm incorporated with clay particles showed improved tensile and young’s modulus properties. Similar tests were reported by Arrakhiz et al. In this work, they reported the effect of mechanical and thermal properties of pine fiber reinforced with clay particles hybrid composites [124]; the results were similar to those of the previous research. The addition of clay particles leads to the elimination the voids in the composite surface and adversely affect the surface properties and overall performance of the composite.

Nanoclay particles are one of the most commonly used filler materials due to its availability and inexpensiveness. It comes under the silicate group nanomaterials. Nanoclay particles in the composite material can increase the mechanical, physical and chemical properties. The size and shape of the particles also plays an important role in the composite material for the strength and bonding between reinforcement and matrix material. Toyota automobile industries first started using the nanoclay particles in the polymer composite fabrication [125]; in this research work, the author has shown the importance of clay particles as a filler material to create the interfacial bond between the matrix and reinforcement and an improvement in the physical and fracture toughness of the composite material. Due to advancements in the material properties by using the nanoclay particles, the materials have been used in several functional and structural applications. The microstructure of the nanoparticles present in the material plays a key role in the improvement of mechanical properties.

Generally, nanoclays are available in several forms such as nanoclay platelets, calcined nanoclay and natural montmorillonite (CB). Assaedi et al. investigated the effect of nanoclay platelets (also called cloisite 30B) on the thermal and mechanical properties of fly ash geopolymer [126]. In this study, the platelets are added by varying the weight percentages such as 1%, 2% and 3% loadings. Results showed that adding the nanoclay platelets to the geopolymer shows a slight increase in mechanical properties. Flexural strength and flexural modulus were increased significantly with an increase in nanoclay loading up to 2% weight loading. After 2% weight, the properties of the materials slightly decreased due to porosity and poor dispersion. It is evident that continuous adding of the filler materials to the geopolymers does not increases its mechanical properties. On the other hand, Assaedi also stated the conditions for the temperature variant; he studied this by using the thermo-gravimetric analysis in terms of weight. It was observed that most of the weight loss occurred at 3% nanoclay loading due to extra friction.

Hakamy et al. studied the effect of calcined nanoclay (CNC) on mechanical and microstructural properties of hemp fiber reinforced Portland cement nanocomposites [127]. Calcined nanoclay was prepared by using the heat treatment process of nanoclay particles at 800, 850 and 900 °C temperature for 2 h. It was found that nanoclay converted into a calcined state at 900 °C. Experimental results suggest that the mechanical properties, such as flexural strength and fracture toughness, were improved by adding the CNC to the composite material. Calcined nanoclay particles were added by varying its weight percentages such as 1%, 2% and 3%. The optimum hemp fabric content was found as 6.9 % and CNC was found as 1%.

Eesaee et al. worked on the effect of nanoclay particles in the reinforced glass/woven fiber composites with phenolic resins [128] and mainly dealt with the natural montmorillonite (CN) and artificial montmorillonite (CB). The filler material improved the composite performance because of the proper dispersion of nanoclay particles on the composite surface, which lead to better interfacial bonding with the matrix and reinforcement. It was exposed that the amalgamation of 2.5 wt.% of the filler material boosts the elastic modulus up to 38% for CN and 43% for CB. On the other side, aging with water molecules and hydroxyl groups of the phenolic matrix leads to a decrease in the strength of the composite.

Avil et al. observed the effect of porosity and failure strength in the presence of nanoclay in glass fiber reinforced epoxy composites [129]. The results showed that the additional filler material caused the reduction of porosity by 73.2% at 10 wt.%. Binu et al. studied the effect of nanoclay, cloisite 15A, on the mechanical performance and thermal properties of polyester-based glass fiber composites [130]. The results showed a maximum mechanical performance observed at 1% loading of the filler materials to the composite. According to this study, the glass transmission temperature failed in between 95 and 110 °C. John et al. observed the dynamic mechanical and thermal properties of cyanate ester syntactic foams besides the nanoclay material [131]. The studies showed that a significant improvement in mechanical properties like tensile, flexural strength moduli was detected for the foams on the integration of nanoclay particles. Nanoclay hikes the toughness of the overall composite and, on the other hand, thermal properties are not much prejudiced by the addition of filler materials.

Maharsia et al. [132] studied mechanical properties in syntactic foams through nanoclay reinforcement. Results indicated that the presence of nanoclay (2% and 5% by weight) showed an improvement in mechanical properties though there was a reduction in modulus due to improper bonding between nanoclay particles and matrix material. Withers et al. [133] observed the effect of cloisite 25A montmorillonite (MMT) nanoclay filler material in epoxy-based composites. The nanocomposites were fabricated by varying the weight percentages of nanoparticles in it. According to the results, the optimum mechanical properties are shown at 2 wt.% of MMT clay addition. The improved surface properties are observed at the low percentage loadings of MMT clay particles. Mo-lin Chan et al. [134] studied the mechanism of reinforcement in nanoclay polymer composite. Different mechanical tests were conducted by varying the content of nanoparticles in the composite material. Interestingly the results are showed that tensile strength and modulus of the composite increased by 34% and 25% correspondingly. The establishment of limitations between the nanoclay clusters and epoxy resin can filter the matrix grains and additionally improve the strength of the composite. Some of the research publications on nanofillers in the composite materials are discussed in Table 14.

## 7. Applications

Fiber-reinforced composites are better than pure polymer composites. There should be an occurrence of auxiliary applications on account of its high mechanical properties and lower expense. Due to an account of advanced properties, hybrid composites are a replacement for synthetic composites, particularly when it comes to the load-bearing and construction applications [24]. A recent study stated that goods made up of natural fiber composites have been increased and used in several applications such as ward boards, windows, car door panels, tables, packing selves and lightweight goods [156]. The utilization of natural fiber composites in place of synthetic and petroleum-based fiber composites has increased drastically in various sectors such as sports equipment, aerospace, office machinery, automobile, and shipping industries are one of the main application sectors for lightweight composite materials. The researchers found interest in the replacement of bio fiber-based composites due to its abundance, cost-effectiveness, recyclability, ecofriendliness, low fabrication cost and biodegradability [157,158].

A recent survey stated that the fabrication of fiber-reinforced polymer composites has become more popular in commercial applications, especially in the aerospace industry. The utilization of fiber-based composite caused a reduction in overall weight by 35% of the aircraft, and shows a positive effect on fuel consumption and efficiency of the overall performance. Due to these properties, aircraft industries such as Boeing, Dreamliner and Airbus companies have started using the composites in the majority of their components [159]. The main crucial factor in composite fabrication is weight reduction. Some of the natural fibers serve as a natural filler material in the composite fabrication in the presence of bio-based and biodegradable matrix material designed for the extrusion film blowing of thin packages [160].

Santos da luz et al. worked on natural fiber composites in the replacement of synthetic fiber composites for military applications [161]. They compared natural fiber (pineapple) epoxy composite material and synthetic (aramid) fiber composite material in a hard armor. Results showed that the armor plate made from natural fibers has a stronger impact strength than synthetic fiber plates. Natural fiber hybrid nanocomposites are used in various industries because of its improvement in mechanical, thermal and moisture absorption along with light weight and less cost. Nowadays, these composites are in construction, biomedical, filtration, drug delivery, tissue template, wound dressing and cosmetics. Some of the potential applications are listed below [162].

1. Filter media: Liquid, gas and molecule filtration.
2. Nano-sensors: Biochemical, thermal and piezoelectric sensor.
3. Cosmetics: Skin cleaning and healing.
4. Life science: Drug delivery carrier and wound dressing.
5. Tissue engineering scaffolding: Porous membrane for skin, 3D scaffolds for bone and cartilage regeneration.
6. Industrial applications: Micro/nano electronic devices, electrostatic dissipation, LCD devices, lightweight space craft materials.

## 8. Conclusions and Future Work

In this review paper, fiber-based hybrid composites have been evaluated, specifically on account of their properties such as surface morphology, thermal stability, dynamic mechanical, mechanical, moisture absorption, fire retardance and applications. Hybridization became famous, not just because of the improved presentation of the subsequent items, but also due to the possibility of conquering the confinements that hamper the relevance of common filaments in specialized structures. There is an immense chance that a huge level of natural fibers can be consolidated in ordinary engineered fortified composites items, which is a major advance from biological and affordable perspectives. The quality natural fibers rely on the development conditions and development time affects the performance of the composite. Although there are so many advantages to using natural fibers in commercial applications, it is bounded to some of the applications because of its biodegradability and flammable properties compared to synthetic fiber composites. To overcome this problem, the researchers started to work on chemical treatment of natural fibers under various conditions. These modifications improved the fiber strength and also bonding capacity between matrix and reinforcement. On the other hand, the researchers found an interest in the integration of nanofillers into composite materials. The addition of nanoparticles to the hybrid composites played a crucial role in advancing their properties. The surface properties, fracture toughness, void elimination, amount of water absorbed is decreased and the dynamic mechanical properties are increased because of these nanofiller materials in the hybrid composite. It is worth saying that hybrid composite materials bring a revolution in the real-life commercial applications such as biomedical, aerospace, shipping industry, automobile, biomedical, construction, drug delivery, wound dressing and gas filtration applications. In the future, the alterations in fabrication techniques and the addition of nanofillers may lead to improvement as well as prediction of the results is possible, which attracts researchers to use it in multiple applications.

## Figures and Tables

**Table 1:** Natural fibers chemical composition [22,24,25,26].

Fiber Type	Cellulose (%)	Hemi-Cellulose (%)	Lignin (%)	Pectin (%)	Wax (%)	Ash (%)	Moisture (%)	Microfibrillar-Angle (°)
Abaca	56–63	20–25	7–9	-	3	-	-	20–25
Bamboo	26–43	30	1–31	-	10	-	9.16	-
Banana	63–83	-	5	-	11	-	10.71	11–12
Coir	36–43	41–45	0.15–0.25	3–4	-	-	11.36	30–49
Cotton	83–91	3	-	0.6	8–9	-	7.85–8.5	-
Flax	64–72	64–72	2–2.2	1.8–2.3	-	-	8–12	5–10
Hemp	70–74	0.9	3.7–5.7	0.8	1.2–6.2	0.8	6.2–12	2–6.2
Jute	61–72	18–22	12–13	0.2	0.5	0.5–2	12.5–13.7	8
Kenaf	45–57	8–13	22	0.6	0.8	2–5	6.2–12	2–6.2
Nettle	86	5.4	4.0	0.6	3.1	-	-	-
Rachis	43	-	26	-	-	-	-	28–37
Ramie	69–91	5–15	0.4–0.7	1.9	-	-	-	69–83
Rice husk	38–45	-	-	-	-	20	-	-
Sisal	78	10	8	-	2	1	11	-
Hardwood	38–49	17–23	23–30	-	-	-	-	-
Softwood	40–45	19–22	26–34	-	-	-	-	-

**Table 2:** Natural fibers origin, physical and mechanical properties [22,26,27,28].

Fiber Type	Origin	Diameter (µm)	Density g/cm^3^	Tensile Strength (MPa)	Young’s Modulus (GPa)	Elongation (%)	Specific Strength (S/ρ)	Specific Modulus (E/ρ)
Abaca	Leaf	-	1.5	430–813	31.1–33.6	2.9–10	267	8
Bagasse	Grass	-	0.89	350	22	5.5	-	-
Bamboo	Grass	240–330	0.6–1.25	290	11–17	-	454	32.6
Banana	Leaf	50–250	1.35	529–914	27–32	2.6–5.9	444	13.2
Coir	Fruit	-	1.15–1.25	131–220	4–6	15–40	146	3.3–5
Cotton	Seed	-	1.51	400	12	3–10	179–373	3.44–7.9
Flax	Stem	-	1.4	800–1500	60–80	1.2–1.6	535–1000	18.4–53
Hemp	Stem	-	1.48	550–900	70	1.6–4.0	372–608	47.3
Jute	Stem	40–350	1.31–1.48	393–800	13–26.5	1.16–1.8	269–548	6.85–20.6
Kenaf	Stem	70–250	1.4	284–930	21–60	1.6	641	36.55
Nettle	Bast	-	1.51	650	38	1.7	-	87–115
Pineapple	Leaf	-	1.44	413–1627	60–82	14.5	-	-
Ramie	Stem	50	1.5	500	44	2	147–675	29.3–85
Sisal	Leaf	50–300	1.3–1.4	390–450	12–41	2.3–2.5	366–441	6.5–15.2
Softwood	Wood	-	1500	1000	40	-	667	26.67
Hardwood	Wood	-	1200	950	37.9	-	-	-

**Table 3:** Merits and demerits of natural fibers.

Merits	Demerits
Lightweight	Flammable
Recyclable	Dimensional instability
Improved specific mechanical properties	High moisture absorption
Eco-friendly	Anisotropic behavior
Do not generate any harmful gasses during processing	Limited processing temperature (~200–230 °C)
Good thermal properties	Sensitive to UV
Good acoustic properties	Fugal attack and microbial
Low cost, availability	Low strength than synthetic fibers

**Table 4:** Mechanical properties of synthetic fibers [26,27,28].

Type of Fiber	Density (g/cm^3^)	Tensile Strength (MPa)	Young’s Modulus (GPa)	Elongation (%)
Aramid	1.44	3000	124	2.5
Carbon	1.4	400	230–240	1.4–1.8
E-Glass	2.55	2000–3500	63–67	2.5
S-Glass	2.5	4570	70–73	1.8–3.2
Silicon carbide	3.16	-	360–440	-

**Table 5:** Advantages and disadvantages of synthetic fibers.

Advantages	Disadvantages
Long lasting	Flammable
Readily pick-up to various dyes	Prone to heat damage
Stretchable	Melt easily
Waterproofing	Not eco-friendly
Non biodegradability	Cause for microplastic pollution
Moisture resistance	Not suitable for hot washing
Strain and wear resistance	Poor insulation capacity

**Table 6:** Chemical treatment processes and its importance on hybrid fiber composites.

Chemical Treatment	Fibers	Advantages	Reference
Alkaline treatment	Flax Jute Sisal	Improves the surface roughness Better mechanical interlocking, and cellulose content.	[32,33,34]
Silane treatment	Glass	Prevent swelling of composite Improve thermal stability.	[35,36]
Acetylation treatment	Sisal	Improve the bio-resistance of a composite and fiber-matrix interactions.	[37,38]
Benzoylation treatment	Flax Sisal	Reduces the moisture absorption, improve its thermal stability and strength.	[39,40,41]
Acrylonitrile grafting and Acrylation	Oil palm Glass	It improves the tensile strength and young’s modulus.	[42]
Malleated coupling gents	Cellulose fibers Banana Hemp	Growth in flexural strength, hardness, impact strength and flexural modulus.	[43,44]
Permanganate treatment	Cellulose fibers	It improves the hydrophilic tendency of the composite.	[45]
Peroxide treatment	Oil palm	Improves tensile properties.	[45]
Isocyanate treatment	Leaf fibers	Enhance better mechanical properties.	[46,47]

**Table 7:** Thermoplastic resins advantages and disadvantages.

Type of Resin	Advantages	Disadvantages
Polystyrene (PS)	Moisture resistance Weather resistance Good chemical resistance	Flammable Brittle Low impact resistance
Polypropylene (PP)	Good fatigue resistance High thermal resistance High dielectric resistance Excellent chemical resistance	Hard to process Comparatively high cost Limited availability
Polyethylene (PE)	Low cost Lightweight Low moisture absorption Good fatigue resistance Good impact strength High ductility	Flammable High thermal expansion Poor weather resistance
Polyvinyl chloride (PVC)	Versatility High impact strength Fire retardant Advance in chemical resistance Less cost Excellent dimensional stability	Poor resistance to UV and temperature
Natural rubber	Good tensile strength High resilience Tear resistance Low cost Water resistance	Poor resistance to hydrocarbons Chances for swelling Complete dissolution

**Table 8:** Mechanical properties of thermoplastic resins.

Polymer	Density (g/cm^3^)	Tensile Strength (MPa)	Young’s Modulus (GPa)	Elongation (%)	Glass Transition Temp.(°C)
Polyethylene (PE)	0.9	18.0	0.5	350.0	−78.0
High-density polyethylene (HDPE)	0.9–1.0	32.0–38.2	1.3	150.0	−110.0
Low-density polyethylene (LDPE)	0.9	10.0–11.6	0.2–0.3	400.0	−110.0
Polyethylene terephthalate (PET)	1.5–1.6	55.0–159.0	2.3–9.0	300.0	67.0
Polystyrene (PS)	1.04	34.0	3.0	1.6	100.0
High-impact polystyrene (HIPS)	1.0	42.0	2.1	2.5	88.0–92.0
Polycarbonate (PC)	1.2	69.0	2.3	200.0	147.0
Polypropylene (PP)	0.9–1.3	35.8	1.6	80.0	−20.0
Poly methyl methacrylate (PMMA)	1.1–1.2	72.4	3.0	2.5	125.0
Polyvinyl chloride (PVC)	1.3–1.5	52.0–90.0	3.0–4.0	50.0–80.0	82.0
Nylon 6 (PA 6)	1.1	81.4	2.8	60.0	47.0
Nylon 6,6 (PA 6,6)	1.1	82.7	2.8	60.0	70.0

**Table 9:** Recent research on thermoplastic hybrid composites and fabrication techniques.

Matrix	Reinforcement	Fabrication Process	Properties Observed	Reference
Polyethylene (PE)	Coir fibers	Rotational molding	Water absorption SEM Mechanical measurements	[66]
Polyethylene (PE)	Cupula fibers	Screw extruder	Tensile, flexural properties Thermogravimetric Differential scanning calorimetry	[67]
Polyolefin	Coir coconut fibers	Injection molding	Mechanical Viscoelastic Thermal properties	[68]
LDPE	Pineapple fibers	Compression molding	Flexural and Izod impact Water absorption Thermal resistance	[69]
LDPE	Jute fibers	Compression molding	Mechanical properties Moisture intake	[70]
HDPE	Jute fibers	Sandwich technique	Fracture morphology Mechanical performance	[71]
HDPE	Oil-palm	Injection molding	Thermal properties Dynamic mechanical properties	[72]
PET	Moringa olefin fruit fibers	Hot press	Thermal Mechanical Morphological	[73]
PET	Hemp	Vacuum infusion	Flexural Tensile Water absorption	[74]
HIPS	Sisal	Compression molding	Tensile Fracture	[75]
Polycarbonate (PC)	Carbon fiber	Injection molding	Thermomechanical Tensile Flexural Izod	[76]
Polypropylene	Kenaf Pineapple	Hot press molding compression	Tensile Flexural Water absorption Surface morphology	[77]
Polypropylene	Flax	Hot press technique	Water absorption Sound transmission loss test Tensile Microscopic analysis	[78]
Polypropylene	Coir fibers	Melt-blending	Friction and wear	[79]
Polypropylene	Banana glass	Twin-screw extruder	Tensile Flexural Impact Water absorption	[80]
PVC	Wood flour Zinc borate	Twin screw extruder + melt press	Thermal properties Mechanical properties	[81]
Polypropylene	Marble waste	Injection molding	Moisture absorption Surface morphology Mechanical properties	[82]

**Table 10:** Merits and demerits of thermoset resins.

Type of Resin	Advantages	Disadvantages
Epoxy resin	High water resistance High thermal and mechanical properties Low curing time Durability	Difficult to process Corrosive amine hardener More expensive than vinyl ester
Vinyl ester	High chemical resistance High mechanical properties than polyester	High curing shrinkage High styrene content Needs post curing More expensive
Polyester	Lowest cost Easy to use	Limited access moderate mechanical properties Huge curing shrinkage High gas emissions
Phenolic	High fire retardance	Hard to develop

**Table 11:** Mechanical properties of thermoset resins.

Polymer	Density (g/cm^3^)	Tensile Strength (MPa)	Young’s Modulus (GPa)	Elongation (%)	Glass Transition Temp. (°C)
Epoxy resin (EP)	1.2–1.3	600.0	80.0	1.3	70.0–167
Urea formaldehyde (UF)	1.5–1.6	65.0	9.0	0.8	130.0
Melamine formaldehyde (MF)	1.5–1.6	65.0	12.0	0.6	150.0
Phenol formaldehyde (PF)	1.2	45.0	6.5	1.2	170.0
Unsaturated polyester (UPE)	1.1	60.0	3.4	2.0	110.0–188.0
Rigid thermoset polyurethane (RPU)	1.2	60.0	2.2	90.0	−150.0–0.0

**Table 12:** Recent research on thermoplastic hybrid composites and fabrication techniques.

Matrix	Reinforcement	Fabrication Process	Properties Observed	Reference
Epoxy	Hemp fibers Flax fibers	Hand layup	Mechanical Moisture analysis Contact angle measurement	[98]
Epoxy	Jute fibers glass fibers	Vacuum infusion	Compressive strength Shear strength	[90]
Epoxy	Crab shell Sisal fibers	Compression molding	Mechanical Chemical Morphological	[99]
Epoxy	Woven cotton Bamboo	Compression molding	Flexural Tensile Impact	[100]
Epoxy	Basalt fiber UHMWPE	Compression molding	Impact Tensile Compression	[101]
Epoxy	Glass Basalt woven	Resin transfer molding technique	Mechanical properties Impact behavior	[102]
Epoxy	Pineapple Basalt	Compression molding	Mechanical properties Tensile Flexural Damping	[103]
Epoxy	Sisal Banana Coir	Compression molding	Hardness Tensile Flexural Water absorption	[104]
Epoxy	Kevlar Cocos nucifera	Compression molding + hand layup	Thermal Mechanical	[105]
Epoxy	Carbon Flax	Hand layup	Impact strength	[106]
Epoxy	Jute Basalt	Hand layup	Mechanical properties Water absorption	[107]
Epoxy	Bamboo Kenaf	Hand layup	Thermal properties	[108]
Epoxy	Banana Flax	Hand layup	Tensile strength Flexural strength Thermal properties	[109]
Epoxy	Neem Banyan leaf	Hand layup	Thermal stability Thermogravimetry	[110]
Epoxy	Coir Kevlar	Hand layup	Mechanical properties	[111]
Vinyl ester	Glass	The Electroless plating method	Thermal wear analysis Mechanical properties	[112]
Vinyl ester	Flax Basalt	Resin infusion and hand layup	Impact strength Mechanical characteristics	[113]
Vinyl ester	Flax Basalt	Vacuum assisted resin infusion	Interlaminar strength Fracture toughness	[114]
Vinyl ester	Flax Basalt	Vacuum infusion	Fracture toughness Water absorption	[115]
Epoxy	Napier Glass	Cold Pressing	Tensile properties	[116]
Epoxy resin	Sisal Coir	Cold Pressing	Moisture absorption Fire retardation	[117]
Epoxy resin	Long kenaf Woven glass	Cold Pressing	Fracture toughness Water absorption	[118]
PLA	Sisal Hemp fibers	Injection molding+ Extrusion	Mechanical properties Thermal stability	[119]

**Table 13:** Merits and demerits of bio-based resins.

Resin	Merits	Demerits
Cellulose	Inexpensive Easily available Ease to adjust Intermediate impact resistance Intermediate heat resistance Eco-friendly	Relative low decomposition High moisture absorption
Polyhydroxy alkenoates (PHA)	Biodegradable High molecular weight	Low stability Fragile Low decomposition costly
Polylactic acid	Nontoxic nature High strength High modulus Low cost	Brittle Poor impact strength Low thermal degradation
Starch	Low cost Biodegradable	Hard to process Water sensitive Brittle

**Table 14:** Various research publications on nanofillers in fiber composite materials.

Fibers/Nanofillers	Polymer	Investigations	Reference
Glass fibers/Silica	Epoxy resin	Elastic properties	[135]
Pineapple/Silica	Epoxy resin	Tensile fatigue Fracture toughness	[136]
Rubber/Silica	Epoxy resin	Vibration and damping	[137]
Glass/Kevlar/Silica	Epoxy resin	Fracture toughness Tensile strength Flexural strength	[138]
Banana/Sisal/Glass/Redmud	Polyester resin	Tensile strength Flexural strength Impact strength	[139]
Silica/Glass/Graphite	Polypropylene	Thermo Mechanical properties and elastic properties	[140]
Basalt/Clay	Polypropylene	Impact Properties	[141]
Carbon fiber/Carbon nanotube	Polyethylene	Mechanical properties	[142]
Rubber/Carbon nanotube	--	Stress-strain analysis	[143]
Carbon fiber/Carbon nanotube	--	Mechanical properties	[144]
Bamboo/Clay	Polyvinyl alcohol	Thermal, Physical and mechanical properties	[145]
Pineapple/Carbon/Jute/Watermelon peel nano particles form	Epoxy resin	Tensile, flexural and fracture toughness	[146]
Sugar palm/Clay	Polyester	Thermal and mechanical properties	[147]
Glass/Clay	Polyester	Mechanical properties	[148]
Glass/Clay	Vinyl ester	Thermal and flame retardancy studies	[149]
Glass/Silica	Vinyl ester	Mechanical properties	[150]
Glass/Clay	Vinyl ester	Mechanical, thermal and vibration properties	[151,152,153]
Glass/Clay	Vinyl ester	Mechanical properties	[154]
Basalt/graphene oxide	Epoxy resin	Interlaminar shear strength Fracture toughness	[155]

## References

[1] Megahed M., Abo-bakr R.M., Mohamed S.A. (2020). Optimization of hybrid natural laminated composite beams for a minimum weight and cost design. Compos. Struct..

[2] Wambua P., Ivens J., Verpoest I. (2003). Natural fibers: Can they replace glass in fiber reinforced plastics. Compos. Sci. Technol..

[3] Rajak D.K., Pagar D.D., Menezes P.L., Linul E. (2019). Fiber-Reinforced Polymer Composites: Manufacturing, properties, and applications. Polymers.

[4] Saba N., Paridah M.T., Abdan K., Ibrahim N.A. (2016). Dynamic mechanical properties of oil palm nano filler/kenaf/epoxy hybrid nanocomposites. Constr. Build. Mater..

[5] Ahmad F., Choi H.S., Park M.K. (2015). A review: Natural fiber composites selection in view of mechanical, light weight and economic properties. Macromol. Mater. Eng..

[6] Kiruthika A.V. (2017). A review on physico-mechanical properties of bast fibre reinforced polymer composites. J. Build. Eng..

[7] George J., Sreekala M.S., Thomas S. (2001). A review on interface modification and characterization of natural fiber reinforced plastic composites. Polym. Eng. Sci..

[8] Faruk O., Bledzki A.K., Fink H.P., Sain M. (2012). Biocomposites reinforced with natural fibers: 2000–2010. Prog. Polym. Sci..

[9] Biswas B., Kandola B.K. (2011). The effect of chemically reactive type flame retardant additives on flammability of PES toughened epoxy resin and carbon fiber-reinforced composites. Polym. Adv. Technol..

[10] John M.J., Anandjiwala R.D. (2008). Recent developments in chemical modification and characterization of natural fiber-reinforced composites. Polym. Compos..

[11] Galos J. (2020). Thin-ply composite laminates: A review. Compos. Struct..

[12] Grefe H., Kandula M.W., Dilger K. (2020). Influence of the fibre orientation on the lap shear strength and fracture behavior of adhesively bonded composite metal joints at high strain rates. Int. J. Adhes. Adhes..

[13] Ou Y., Gattas J.M., Fernando D., Torero J.L. (2020). Experimental investigation of a timber-concrete floor panel system with a hybrid glass fibre reinforced polymer-timber corrugated core. Eng. Struct..

[14] Mishra S., Mohanty A.K., Drzal L.T., Misra M., Parija S., Nayak S.K., Tripathy S.S. (2003). Studies on mechanical performance of biofibre/glass reinforced polyester hybrid composites. Compos. Sci. Technol..

[15] Jawaid M., Abdul Khalil H.P.S. (2011). Cellulosic/synthetic fibre reinforced polymer hybrid composites: A review. Carbohydr. Polym..

[16] John M.J., Thomas S. (2008). Biofibres and biocomposites. Carbohydr. Polym..

[17] Bhoopathi R., Ramesh M., Deepa C. (2014). Fabrication and property evaluation of banana-hemp-glass fiber reinforced composites. Procedia Eng..

[18] Alavudeen A., Rajini N., Karthikeyan S., Thiruchitrambalam M., Venkateshwaren N. (2015). Mechanical properties of banana/kenaf fiber-reinforced hybrid polyester composites: Effect of woven fabric and random orientation. Mater. Des..

[19] Ramesh M., Palanikumar K., Reddy K.H. (2013). Mechanical property evaluation of sisal-jute-glass fiber reinforced polyester composites. Compos. Part B.

[20] Almeida Junior J.H.S., Amico S.C., Botelho E.C., Amado F.D.R. (2013). Hybridization effect on the mechanical properties of curaua/glass fiber composites. Compos. Part B.

[21] Velmurugan R., Manikandan V. (2007). Mechanical properties of palmyra/glass fiber hybrid composites. Compos. Part A Appl. Sci. Manuf..

[22] Kumar A., Singh R.C., Chaudhary R. (2020). Recent progress in production of metal matrix composites by stir casting process-An overview. Mater. Today Proc..

[23] Rajak D.K., Pagar D.D., Kumar R., Pruncu C.I. (2019). Recent progress of reinforcement materials: A comprehensive overview of composite materials. J. Mater. Res. Technol..

[24] Sharma A.K., Bhandari R., Aherwar A., Rimasauskiene R. (2020). Matrix materials used in composites: A comprehensive study. Mater. Today Proc..

[25] Bharath K.N., Basavarajappa S. (2016). Applications of biocomposite materials based on natural fibers from renewable resources: A review. Sci. Eng. Compos. Mater..

[26] Djafari Petroudy S.R. (2017). 3-Physical and mechanical properties of natural fibers. Adv. High Strength Nat. Fibre Compos. Constr..

[27] Mohanty A.K., Misra M., Hinrichsen G. (2000). Biofibres, biodegradable polymers and biocomposites: An overview. Macromol. Mater. Eng..

[28] Gholampour A., Ozbakkaloglu T. (2020). A review of natural fiber composites: Properties, modification and processing techniques, characterization, applications. J. Mater. Sci..

[29] Wang X., Xie J., Zhang H., Zhang W., An S., Chen S., Luo C. (2016). Determining the lignin distribution in plant fiber cell walls based on chemical and biological methods. Cellulose.

[30] Sood M., Dwivedi G. (2018). Effect of fiber treatment on flexural properties of natural fiber reinforced composites: A review. Egypt. J. Pet..

[31] Li X., Tabil L.G., Panigrahi S. (2007). Chemical treatments of natural fiber for use in natural fiber-reinforced composites: A review. J. Polym. Environ..

[32] Mohanty A.K., Misra M., Drzal L.T. (2001). Surface modifications of natural fibers and performance of the resulting biocomposites: An overview. Compos. Interfaces.

[33] Ray D., Sarkar B.K., Rana A.K., Bose N.R. (2001). Effect of alkali treated jute fibres on composite properties. Bull. Mater. Sci..

[34] Mishra S., Misra M., Tripathy S.S., Nayak S.K., Mohanty A.K. (2001). Graft Copolymerization of Acrylonitrile on Chemically Modified Sisal Fibers. Macromol. Mater. Eng..

[35] Ali A., Shaker K., Nawab Y., Jabbar M., Hussain T., Militky J., Baheti V. (2018). Hydrophobic treatment of natural fibers and their composites—A review. J. Ind. Text..

[36] Rong M.Z., Zhang M.Q., Liu Y., Yang G.C., Zeng H.M. (2001). The effect of fiber treatment on the mechanical properties of unidirectional sisal-reinforced epoxy composites. Compos. Sci. Technol..

[37] Kumar R., Obrai S., Sharma A. (2011). Chemical modifications of natural fiber for composite material. Pelagia Res. Libr..

[38] Valadez-Gonzalez A., Cervantes-Uc J.M., Olayo R., Herrera-Franco P.J. (1999). Effect of fiber surface treatment on the fiber-matrix bond strength of natural fiber reinforced composites. Compos. Part B Eng..

[39] Joseph P.V., Mathew G., Joseph K., Groeninckx G., Thomas S. (2003). Dynamic mechanical properties of short sisal fibre reinforced polypropylene composites. Compos. Part A Appl. Sci. Manuf..

[40] Idicula M., Malhotra S.K., Joseph K., Thomas S. (2005). Dynamic mechanical analysis of randomly oriented intimately mixed short banana/sisal hybrid fibre reinforced polyester composites. Compos. Sci. Technol..

[41] Paul S., Nanda P., Gupta R. (2003). PhCOCI-Py/basic alumina as a versatile reagent for benzoylation in solvent-free conditions. Molecules.

[42] Pinem J.A., Wardani A.K., Aryanti P.T.P., Khoiruddin K., Wenten I.G. (2019). Hydrophilic Modification of Polymeric Membrane using Graft Polymerization Method: A Mini Review. IOP Conf. Ser. Mater. Sci. Eng..

[43] Park J.M., Quang S.T., Hwang B.S., DeVries K.L. (2006). Interfacial evaluation of modified Jute and Hemp fibers/polypropylene (PP)-maleic anhydride polypropylene copolymers (PP-MAPP) composites using micromechanical technique and nondestructive acoustic emission. Compos. Sci. Technol..

[44] Mohanty S., Nayak S.K., Verma S.K., Tripathy S.S. (2004). Effect of MAPP as a Coupling Agent on the Performance of Jute-PP Composites. J. Reinf. Plast. Compos..

[45] Sreekala M.S., Kumaran M.G., Joseph S., Jacob M., Thomas S. (2000). Oil palm fibre reinforced phenol formaldehyde composites: Influence of fibre surface modifications on the mechanical performance. Appl. Compos. Mater..

[46] Suwanruji P., Tuechart T., Smitthipong W., Chollakup R. (2017). Modification of pineapple leaf fiber surfaces with silane and isocyanate for reinforcing thermoplastic. J. Thermoplast. Compos. Mater..

[47] Shih Y.F., Chang W.C., Liu W.C., Lee C.C., Kuan C.S., Yu Y.H. (2014). The inclusion of PALF made the thermoplastic matrix stiffer, especially for the LDPE composites. J. Taiwan Inst. of Chem. Eng..

[48] Sanjay M.R., Madhu P., Jawaid M., Senthamaraikannan P., Senthil S., Pradeep S. (2018). Characterization and properties of natural fiber polymer composites: A comprehensive review. J. Clean. Prod..

[49] Fei M.E., Xie T., Liu W., Chen H., Qiu R. (2017). Surface grafting of bamboo fibers with 1,2-epoxy-4-vinylcyclohexane for reinforcing unsaturated polyester. Cellulose.

[50] Seki Y. (2009). Innovative multifunctional siloxane treatment of jute fiber surface and its effect on the mechanical properties of jute/thermoset composites. Mater. Sci. Eng. A.

[51] Jarukumjorn K., Suppakarn N. (2009). Effect of glass fiber hybridization on properties of sisal fiber-polypropylene composites. Compos. Part B Eng..

[52] Schmidt T.M., Goss T.M., Amico S.C., Lekakou C. (2009). Permeability of hybrid reinforcements and mechanical properties of their composites molded by resin transfer molding. J. Reinf. Plast. Compos..

[53] Nayak S.K., Mohanty S., Samal S.K. (2009). Influence of short bamboo/glass fiber on the thermal, dynamic mechanical and rheological properties of polypropylene hybrid composites. Mater. Sci. Eng. A.

[54] Vega-Hernández M.A., Rosas-Aburto A., Vivaldo-Lima E., Vázquez-Torres H., Cano-Díaz G.S., Pérez-Salinas P., Hernández-Luna M.G., Alcaraz-Cienfuegos J., Zolotukhin M.G. (2019). Development of polystyrene composites based on blue agave bagasse by in situ RAFT polymerization. J. Appl. Polym. Sci..

[55] Haneefa A., Bindu P., Aravind I., Thomas S. (2008). Studies on tensile and flexural properties of short banana/glass hybrid fiber reinforced polystyrene composites. J. Compos. Mater..

[56] Hao X., Zhou H., Mu B., Chen L., Guo Q., Yi X., Sun L., Wang Q., Ou R. (2020). Effects of fiber geometry and orientation distribution on the anisotropy of mechanical properties, creep behavior and thermal expansion of natural fiber/HDPE composites. Compos. Part B Eng..

[57] Robledo-Ortíz J.R., González-López M.E., Rodrigue D., Gutiérrez-Ruiz J.F., Prezas-Lara F., Pérez-Fonseca A.A. (2020). Improving the Compatibility and Mechanical Properties of Natural Fibers/Green Polyethylene Biocomposites Produced by Rotational Molding. J. Polym. Environ..

[58] Paran S.M.R., Naderi G., Shokoohi S., Ebadati J., Dubois C. (2019). Mechanical and Thermal Properties of Green Thermoplastic Elastomer Vulcanizate Nanocomposites Based on Poly (vinyl chloride) and Nitrile Butadiene Rubber Containing Organoclay and Rice Straw Natural Fibers. J. Polym. Environ..

[59] Ji B., Gao H. (2004). Mechanical properties of nanostructure of biological materials. J. Mech. Phys. Solids.

[60] Zare Y., Garmabi H. (2015). A developed model to assume the interphase properties in a ternary polymer nanocomposite reinforced with two nanofillers. Compos. Part B Eng..

[61] Boussehel H., Mazouzi D.E., Belghar N., Guerira B., Lachi M. (2019). Effect of chemicals treatments on the morphological, mechanical, thermal and water uptake properties of polyvinyl chloride/palm fibers composites. Rev. Compos. Mater. Av..

[62] Ihamouchen C., Djidjelli H., Boukerrou A., Krim S., Kaci M., Martinez J.J. (2012). Effect of surface treatment on the physicomechanical and thermal properties of high-density polyethylene/olive husk flour composites. J. Appl. Polym. Sci..

[63] Kaci M., Djidjelli H., Boukerrou A., Zaidi L. (2007). Effect of wood filler treatment and EBAGMA compatibilizer on morphology and mechanical properties of low-density polyethylene/olive husk flour composites. Express Polym. Lett..

[64] Haseena A.P., Dasan K.P., Namitha R., Unnikrishna G., Thomas S.A. (2004). Investigation on interfacial adhesion of short sisal/coir hybrid fibre reinforced natural rubber composites by restricted equilibrium swelling technique. Compos. Interfaces.

[65] Prasantha Kumar R., Thomas S. (2001). Interfacial adhesion in sisal fiber/SBR composites: An investigation by the restricted equilibrium swelling technique. J. Adhes. Sci. Technol..

[66] Sari P.S., Thomas S., Spatenka P., Ghanam Z., Jenikova Z. (2019). Effect of plasma modification of polyethylene on natural fibre composites prepared via rotational moulding. Compos. Part B Eng..

[67] Boran Torun S., Pesman E., Donmez Cavdar A. (2019). Effect of alk ali treatment on composites made from recycled polyethylene and chestnut cupula. Polym. Compos..

[68] Salazar M.A.H., Aguirre J.P.C., Navarro S.G., Blay L.R. (2020). Injection molding of coir coconut fiber reinforced polyolefin blends: Mechanical, viscoelastic, thermal behavior and three-dimensional microscopy study. Polymers.

[69] Rahman H., Alimuzzaman S., Sayeed M.M.A., Khan R.A. (2019). Effect of gamma radiation on mechanical properties of pineapple leaf fiber (PALF)-reinforced low-density polyethylene (LDPE) composites. Int. J. Plast. Technol..

[70] Sahadat Hossain M., Razzak M., Uddin M.B., Chowdhury A.M.S., Khan R.A. (2019). Physico-mechanical properties of jute fiber-reinforced LDPE-based composite: Effect of disaccharide (sucrose) and gamma radiation. Radiat. Eff. Defects Solids.

[71] Sayem A.S.M., Haider J., Sayeed M.A. (2019). Development and characterisation of multi-layered jute fabric-reinforced HDPE composites. J. Compos. Mater..

[72] Essabir H., Boujmal R., Bensalah M.O., Rodrigue D., Bouhfid R., Qaiss A.E.K. (2016). Mechanical and thermal properties of hybrid composites: Oil-palm fiber/clay reinforced high density polyethylene. Mech. Mater..

[73] Nayak S., Kumar Khuntia S. (2019). Development and study of properties of Moringa oleifera fruit fibers/polyethylene terephthalate composites for packaging applications. Compos. Commun..

[74] Ahmad M.A.A., Abdul Majid M.S., Ridzuan M.J.M., Mazlee M.N., Gibson A.G. (2018). Dynamic mechanical analysis and effects of moisture on mechanical properties of interwoven hemp/polyethylene terephthalate (PET) hybrid composites. Constr. Build. Mater..

[75] Antich P., Vázquez A., Mondragon I., Bernal C. (2006). Mechanical behavior of high impact polystyrene reinforced with short sisal fibers. Compos. Part A Appl. Sci. Manuf..

[76] Andrzejewski J., Misra M., Mohanty A.K. (2018). Polycarbonate biocomposites reinforced with a hybrid filler system of recycled carbon fiber and biocarbon: Preparation and thermomechanical characterization. J. Appl. Polym. Sci..

[77] Feng N.L., Malingam S.D., Ping C.W., Razali N. (2019). Mechanical properties and water absorption of kenaf/pineapple leaf fiber-reinforced polypropylene hybrid composites. Polym. Compos..

[78] Haris A., Kureemun U., Tran L.Q.N., Lee H.P. (2019). Water Uptake and Its Effects on Mechanical and Acoustic Properties of flax/polypropylene Composite. J. Nat. Fibers.

[79] Liu L., Wang Z., Yu Y., Fu S., Nie Y., Wang H., Song P. (2019). Engineering Interfaces toward High-Performance Polypropylene/Coir Fiber Biocomposites with Enhanced Friction and Wear Behavior. ACS Sustain. Chem. Eng..

[80] Samal S.K., Mohanty S., Nayak S.K. (2009). Banana/glass fiber-reinforced polypropylene hybrid composites: Fabrication and performance evaluation. Polym. Plast. Technol. Eng..

[81] Fang Y., Wang Q., Guo C., Song Y., Cooper P.A. (2013). Effect of zinc borate and wood flour on thermal degradation and fire retardancy of Polyvinyl chloride (PVC) composites. J. Anal. Appl. Pyrolysis.

[82] Bakshi P., Pappu A., Patidar R., Gupta M.K., Thakur V.K. (2020). Transforming marble waste into high-performance, water resistant and thermally insulative hybrid polymer composites for environmental sustainability. Polymers.

[83] Bakare F.O., Skrifvars M., Åkesson D., Wang Y., Afshar S.J., Esmaeili N. (2014). Synthesis and characterization of bio-based thermosetting resins from lactic acid and glycerol. J. Appl. Polym. Sci..

[84] Ail Mutar M., Attab Z.H.A. (2017). Synthesis, Characterization and Properties of New Unsaturated Polyesters Resins Reinforced with Some Fillers (Carbon Nano, Nano TiO2, TiO2 and ZnO) for Composite Application. Int. J. Appl. Nat. Sci. (IJANS).

[85] Mohammad N.A. (2017). Synthesis, Characterization and Properties of the New Unsaturated Polyester Resins for Composite Application. Master’s Thesis.

[86] Yorseng K., Rangappa S.M., Pulikkalparambil H., Siengchin S., Parameswaranpillai J. (2020). Accelerated weathering studies of kenaf/sisal fiber fabric reinforced fully biobased hybrid bioepoxy composites for semi-structural applications: Morphology, thermo-mechanical, water absorption behavior and surface hydrophobicity. Constr. Build. Mater..

[87] Manalo A.C., Aravinthan T., Karunasena W., Islam M.M. (2010). Flexural behaviour of structural fibre composite sandwich beams in flatwise and edgewise positions. Compos. Struct..

[88] Rwawiire S., Tomkova B., Militky J., Jabbar A., Kale B.M. (2015). Development of a biocomposite based on green epoxy polymer and natural cellulose fabric (bark cloth) for automotive instrument panel applications. Compos. Part B Eng..

[89] Pandey J.K., Ahn S.H., Lee C.S., Mohanty A.K., Misra M. (2010). Recent advances in the application of natural fiber-based composites. Macromol. Mater. Eng..

[90] Alves J.L.C., Prado K.S., De Paiva J.M.F. (2019). Compressive and Interlaminar Shear Strength Properties of Biaxial Fibreglass Laminates Hybridized with Jute Fibre Produced by Vacuum Infusion. J. Nat. Fibers.

[91] Asim M., Jawaid M., Paridah M.T., Saba N., Nasir M., Shahroze R.M. (2019). Dynamic and thermo-mechanical properties of hybridized kenaf/PALF reinforced phenolic composites. Polym. Compos..

[92] Asim M., Paridah M.T., Saba N., Jawaid M., Alothman O.Y., Nasir M., Almutairi Z. (2018). Thermal, physical properties and flammability of silane treated kenaf/pineapple leaf fibres phenolic hybrid composites. Compos. Struct..

[93] Liu Y., Wang L., Liu D., Ma Y., Tian Y., Tong J., Senthamaraikannan P., Saravanakumar S. (2019). Evaluation of wear resistance of corn stalk fiber reinforced brake friction materials prepared by wet granulation. Wear.

[94] Singha A.S., Thakur V.K. (2008). Synthesis and characterization of pine needles reinforced RF matrix based biocomposites. E-J. Chem..

[95] Singha A.S., Thakur V.K. (2009). Mechanical, thermal and morphological properties of grewia optiva fiber polymer matrix composites. Polym.-Plast. Technol. Eng..

[96] Singha A.S., Thakur V.K. (2009). fabrication and characterization of H. sabdariffa fiber reinforced green polymer composites. Polym. Plast. Technol. Eng..

[97] Singha A.S., Thakur V.K. (2009). Fabrication and characterization of S. cilliare fiber reinforced polymer composites. Bull. Mater. Sci..

[98] Atmakuri A., Palevicius A., Griskevicius P., Janušas G. (2019). Investigation of mechanical properties of hemp and flax fibers hybrid composites for biomedical applications. Mechanika.

[99] Soundhar A., Kandasamy J. (2019). Mechanical, Chemical and Morphological Analysis of Crab shell/Sisal Natural Fiber Hybrid Composites. J. Nat. Fibers.

[100] Aruchamy K., Pavayee Subramani S., Palaniappan S.K., Sethuraman B., Velu Kaliyannan G. (2020). Study on mechanical characteristics of woven cotton/bamboo hybrid reinforced composite laminates. J. Mater. Res. Technol..

[101] Yang Z., Liu J., Wang F., Li S., Feng X. (2019). Effect of fiber hybridization on mechanical performances and impact behaviors of basalt fiber/UHMWPE fiber reinforced epoxy composites. Compos. Struct..

[102] Sarasini F., Tirillò J., Valente M., Valente T., Cioffi S., Iannace S., Sorrentino L. (2013). Effect of basalt fiber hybridization on the impact behavior under low impact velocity of glass/basalt woven fabric/epoxy resin composites. Compos. Part A Appl. Sci. Manuf..

[103] Doddi P.R.V., Chanamala R., Dora S.P. (2019). Dynamic mechanical properties of epoxy based PALF/basalt hybrid composite laminates. Mater. Res. Express.

[104] Balaji A., Sivaramakrishnan K., Karthikeyan B., Purushothaman R., Swaminathan J., Kannan S., Udhayasankar R., Madieen A.H. (2019). Study on mechanical and morphological properties of sisal/banana/coir fiber-reinforced hybrid polymer composites. J. Braz. Soc. Mech. Sci. Eng..

[105] Naveen J., Jawaid M., Zainudin E.S., Sultan M.T.H., Yahaya R. (2019). Mechanical and moisture diffusion behavior of hybrid Kevlar/Cocos nucifera sheath reinforced epoxy composites. J. Mater. Res. Technol..

[106] Al-Hajaj Z., Sy B.L., Bougherara H., Zdero R. (2019). Impact properties of a new hybrid composite material made from woven carbon fibers plus flax fibers in an epoxy matrix. Compos. Struct..

[107] Prasad L., Saini A., Kumar V. (2019). Mechanical Performance of Jute and Basalt Fiber Geo-grid-Reinforced Epoxy Hybrid Composite Material. J. Nat. Fibers.

[108] Chee S.S., Jawaid M., Sultan M.T.H., Alothman O.Y., Abdullah L.C. (2019). Evaluation of the hybridization effect on the thermal and thermo-oxidative stability of bamboo/kenaf/epoxy hybrid composites. J. Therm. Anal. Calorim..

[109] Srinivasan V.S., Rajendra Boopathy S., Sangeetha D., Vijaya Ramnath B. (2014). Evaluation of mechanical and thermal properties of banana-flax based natural fibre composite. Mater. Des..

[110] Raja T., Anand P. (2019). Evaluation of Thermal Stability and Thermal Properties of Neem/Banyan Reinforced Hybrid Polymer Composite. Mater. Perform. Charact..

[111] Rashid A.H.A., Ahmad R., Jaafar M., Roslan M.N., Ariffin S. (2011). Mechanical properties evaluation of woven coir and kevlar reinforced epoxy composites. Adv. Mater. Res..

[112] Anand G., Alagumurthi N., Elansezhian R., Venkateshwaran N. (2018). Dynamic mechanical, thermal and wear analysis of Ni-P coated glass fiber/Al_2_O_3_ nanowire reinforced vinyl ester composite. Alex. Eng. J..

[113] Fragassa C., Pavlovic A., Santulli C. (2018). Mechanical and impact characterisation of flax and basalt fibre vinylester composites and their hybrids. Compos. Part B Eng..

[114] Almansour F.A., Dhakal H.N., Zhang Z.Y. (2018). Investigation into Mode II interlaminar fracture toughness characteristics of flax/basalt reinforced vinyl ester hybrid composites. Compos. Sci. Technol..

[115] Almansour F.A., Dhakal H.N., Zhang Z.Y. (2017). Effect of water absorption on Mode I interlaminar fracture toughness of flax/basalt reinforced vinyl ester hybrid composites. Compos. Struct..

[116] Fatinah T.S., Abdul Majid M.S., Ridzuan M.J.M., Hong T.W., Amin N.A.M., Afendi M. (2017). Tensile properties of compressed moulded Napier/glass fibre reinforced epoxy composites. J. Phys. Conf. Ser..

[117] Akash G., Gupta K.G., Venkatesha Gupta N.S., Sreenivas Rao K.V. (2017). A study on flammability and moisture absorption behavior of sisal/coir fiber reinforced hybrid composites. IOP Conf. Ser. Mater. Sci. Eng..

[118] Salleh Z., Taib Y.M., Hyie K.M., Mihat M., Berhan M.N., Ghani M.A.A. (2012). Fracture toughness investigation on long kenaf/woven glass hybrid composite due to water absorption effect. Procedia Eng..

[119] Pappu A., Pickering K.L., Thakur V.K. (2019). Manufacturing and characterization of sustainable hybrid composites using sisal and hemp fibres as reinforcement of poly (lactic acid) via injection moulding. Ind. Crops Prod..

[120] Rosamah E., Hossain M.S., Abdul Khalil H.P.S., Wan Nadirah W.O., Dungani R., Nur Amiranajwa A.S., Suraya N.L.M., Fizree H.M., Mohd Omar A.K. (2017). Properties enhancement using oil palm shell nanoparticles of fibers reinforced polyester hybrid composites. Adv. Compos. Mater..

[121] Boccaccini A.R., Erol M., Stark W.J., Mohn D., Hong Z., Mano J.F. (2010). Polymer/bioactive glass nanocomposites for biomedical applications: A review. Compos. Sci. Technol..

[122] Pappu A., Thakur V.K. (2017). Towards sustainable micro and nano composites from fly ash and natural fibers for multifunctional applications. Vacuum.

[123] Majeed K., Jawaid M., Hassan A.A.B.A.A., Bakar A.A., Khalil H.A., Salema A.A., Inuwa I. (2013). Potential materials for food packaging from nanoclay/natural fibres filled hybrid composites. Mater. Des..

[124] Arrakhiz F.Z., Benmoussa K., Bouhfid R., Qaiss A. (2013). Pine cone fiber/clay hybrid composite: Mechanical and thermal properties. Mater. Des..

[125] El-Sheikhy R., Al-Shamrani M. (2017). Interfacial bond assessment of clay-polyolefin nanocomposites CPNC on view of mechanical and fracture properties. Adv. Powder Technol..

[126] Assaedi H., Shaikh F.U.A., Low I.M. (2016). Effect of nano-clay on mechanical and thermal properties of geopolymer. J. Asian Ceram. Soc..

[127] Hakamy A., Shaikh F.U.A., Low I.M. (2015). Effect of calcined nanoclay on microstructural and mechanical properties of chemically treated hemp fabric-reinforced cement nanocomposites. Constr. Build. Mater..

[128] Eesaee M., Shojaei A. (2014). Effect of nanoclays on the mechanical properties and durability of novolac phenolic resin/woven glass fiber composite at various chemical environments. Compos. Part A Appl. Sci. Manuf..

[129] Ávila A.F., Morais D.T.S. (2009). Modeling nanoclay effects into laminates failure strength and porosity. Compos. Struct..

[130] Binu P.P., George K.E., Vinodkumar M.N. (2016). Effect of Nanoclay, Cloisite15A on the Mechanical Properties and Thermal Behavior of Glass Fiber Reinforced Polyester. Procedia Technol..

[131] John B., Nair C.P.R., Ninan K.N. (2010). Effect of nanoclay on the mechanical, dynamic mechanical and thermal properties of cyanate ester syntactic foams. Mater. Sci. Eng. A.

[132] Maharsia R.R., Jerro H.D. (2007). Enhancing tensile strength and toughness in syntactic foams through nanoclay reinforcement. Mater. Sci. Eng. A.

[133] Withers G.J., Yu Y., Khabashesku V.N., Cercone L., Hadjiev V.G., Souza J.M., Davis D.C. (2015). Improved mechanical properties of an epoxy glass-fiber composite reinforced with surface organomodified nanoclays. Compos. Part B Eng..

[134] Chan M.L., Lau K.T., Wong T.T., Ho M.P., Hui D. (2011). Mechanism of reinforcement in a nanoclay/polymer composite. Compos. Part B Eng..

[135] Hassanzadeh-Aghdam M.K., Ansari R., Mahmoodi M.J. (2019). Micromechanical analysis of the elastic response of glass-epoxy hybrid composites containing silica nanoparticles. Mech. Adv. Mater. Struct..

[136] Dinesh T., Kadirvel A., Hariharan P. (2019). Role of nano-silica in tensile fatigue, fracture toughness and low-velocity impact behaviour of acid-treated pineapple fibre/stainless steel wire mesh-reinforced epoxy hybrid composite. Mater. Res. Express.

[137] Huang C.Y., Tsai J.L. (2015). Characterizing vibration damping response of composite laminates containing silica nanoparticles and rubber particles. J. Compos. Mater..

[138] Gokuldass R., Ramesh R. (2019). Mechanical Strength Behavior of Hybrid Composites Tailored by Glass/Kevlar Fibre-Reinforced in Nano-Silica and Micro-Rubber Blended Epoxy. Silicon.

[139] Prabu V.A., Kumaran S.T., Uthayakumar M., Manikandan V. (2018). Influence of redmud particle hybridization in banana/sisal and sisal/glass composites. Part. Sci. Technol..

[140] Pedrazzoli K.D., Pegoretti A. (2014). Hybridization of short glass fiber polypropylene composites with nanosilica and graphite nanoplatelets. J. Reinf. Plast. Compos..

[141] Reza Khalili S.M., Farsani R.E., Soleimani N., Hedayatnasab Z. (2014). Charpy impact behavior of clay/basalt fiber-reinforced polypropylene nanocomposites at various temperatures. J. Thermoplast. Compos. Mater..

[142] Ahmadi M., Ansari R., Rouhi S. (2019). Fracture behavior of the carbon nanotube/carbon fiber/polymer multiscale composites under bending test–A stochastic finite element method. Mech. Adv. Mater. Struct..

[143] Georgantzinos S.K., Giannopoulos G.I., Anifantis N.K. (2009). Investigation of stress-strain behavior of single walled carbon nanotube/rubber composites by a multi-scale finite element method. Theor. Appl. Fract. Mech..

[144] Malekimoghadam R., Icardi U. (2019). Prediction of mechanical properties of carbon nanotube‒carbon fiber reinforced hybrid composites using multi-scale finite element modelling. Compos. Part B Eng..

[145] Adamu M., Rahman M.R., Hamdan S. (2019). Formulation optimization and characterization of bamboo/polyvinyl alcohol/clay nanocomposite by response surface methodology. Compos. Part B Eng..

[146] Balakrishnan S., Krishnaraj C., Raajeshkrishna C.R. (2019). Mechanical characterization of pineapple, watermelon peel nanoparticles reinforced carbon, jute fabric and its hybrid epoxy composites. Mater. Res. Express.

[147] Shahroze R.M., Ishak M.R., Salit M.S., Leman Z., Chandrasekar M., Munawar N.S., Asim M. (2019). Sugar palm fiber/polyester nanocomposites: Influence of adding nanoclay fillers on thermal, dynamic mechanical and physical properties. J. Vinyl Addit. Technol..

[148] Jawahar P., Balasubramanian M. (2006). Influence of nanosize clay platelets on the mechanical properties of glass fiber reinforced polyester composites. J. Nanosci. Nanotechnol..

[149] Zhu H.G., Liu M.Y., Yuen R.K., Leung C.K., Kim J.K. (2014). Thermal performance and flame retardancy studies of vinyl ester and glass fiber reinforced plastic composites containing nanoclay. J. Compos. Mater..

[150] Shaker K., Nawab Y., Saouab A., Ashraf M., Khan A.N. (2018). Effect of silica particle loading on shape distortion in glass/vinyl ester-laminated composite plates. J. Text. Inst..

[151] Chandradass J., Ramesh Kumar M., Velmurugan R. (2008). Effect of clay dispersion on mechanical, thermal and vibration properties of glass fiber-reinforced vinyl ester composites. J. Reinf. Plast. Compos..

[152] Raghavendra N., Murthy H.N., Mahesh K.V., Mylarappa M., Ashik K.P., Siddeswara D.M.K., Krishna M. (2017). Effect of nanoclays on the performance of mechanical, thermal and flammability of Vinylester based nanocomposites. Mater. Today Proc..

[153] Hussain F., Dean D., Haque A., Shamsuzzoha A.M. (2005). S2-Glass/vinylester polymer nanocomposites: Manufacturing, structures, thermal and mechanical properties. J. Adv. Mater..

[154] Park J.Y., Zureick A.H. (2005). Effect of filler and void content on mechanical properties of pultruded composite materials under shear loading. Polym. Compos..

[155] Kim S.H., Park S.J. (2020). Effect of graphene oxide on interfacial interactions and fracture toughness of basalt fiber-reinforced epoxy composites. J. Nanosci. Nanotechnol..

[156] Shalwan A., Yousif B.F. (2013). In state of art: Mechanical and tribological behaviour of polymeric composites based on natural fibres. Mater. Des..

[157] Alkbir M.F.M., Sapuan S.M., Nuraini A.A., Ishak M.R. (2016). Fibre properties and crashworthiness parameters of natural fibre-reinforced composite structure: A literature review. Compos. Struct..

[158] Yang Y., Boom R., Irion B., Van Heerden D.J., Kuiper P., Wit H. (2012). Recycling of composite materials. Chem. Eng. Process. Process Intensif..

[159] Schiffman J.D., Schauer C.L. (2008). A review: Electrospinning of biopolymer nanofibers and their applications. Polym. Rev..

[160] Kharrat F., Khlif M., Hilliou L., Haboussi M., Covas J.A., Nouri H., Bradai C. (2020). Minimally processed date palm (*Phoenix dactylifera* L.) leaves as natural filler and processing aids in poly (lactic acid) composites designed for the extrusion film blowing of thin packages. Ind. Crops Prod..

[161] Luz F.S.D., Garcia Filho F.D.C., Oliveira M.S., Nascimento L.F.C., Monteiro S.N. (2020). Composites with natural fibers and conventional materials applied in a hard armor: A comparison. Polymers.

[162] Huang Z.M., Zhang Y.Z., Kotaki M., Ramakrishna S. (2003). A review on polymer nanofibers by electrospinning and their applications in nanocomposites. Compos. Sci. Technol..

